# Uncooled Thermal Camera Calibration and Optimization of the Photogrammetry Process for UAV Applications in Agriculture

**DOI:** 10.3390/s17102173

**Published:** 2017-09-23

**Authors:** Krishna Ribeiro-Gomes, David Hernández-López, José F. Ortega, Rocío Ballesteros, Tomás Poblete, Miguel A. Moreno

**Affiliations:** 1 Regional Centre of Water Research, University of Castilla-La Mancha, 02071 Albacete, Spain; krishnaribeiro@yahoo.com.br (K.R.-G.); Jose.Ortega@uclm.es (J.F.O.); Rocio.Ballesteros@uclm.es (R.B.); 2 Institute of Regional Development, University of Castilla-La Mancha, 02071 Albacete, Spain; David.Hernandez@uclm.es; 3 Centro de Investigación y Transferencia en Riegoy Agroclimatología, Universidad de Talca, Talca 3460000, Chile; totopoblete@gmail.com

**Keywords:** uncooled thermal camera calibration, microbolometer, unmanned aerial vehicle, image filtering, structure from motion, irrigation management

## Abstract

The acquisition, processing, and interpretation of thermal images from unmanned aerial vehicles (UAVs) is becoming a useful source of information for agronomic applications because of the higher temporal and spatial resolution of these products compared with those obtained from satellites. However, due to the low load capacity of the UAV they need to mount light, uncooled thermal cameras, where the microbolometer is not stabilized to a constant temperature. This makes the camera precision low for many applications. Additionally, the low contrast of the thermal images makes the photogrammetry process inaccurate, which result in large errors in the generation of orthoimages. In this research, we propose the use of new calibration algorithms, based on neural networks, which consider the sensor temperature and the digital response of the microbolometer as input data. In addition, we evaluate the use of the Wallis filter for improving the quality of the photogrammetry process using structure from motion software. With the proposed calibration algorithm, the measurement accuracy increased from 3.55 °C with the original camera configuration to 1.37 °C. The implementation of the Wallis filter increases the number of tie-point from 58,000 to 110,000 and decreases the total positing error from 7.1 m to 1.3 m.

## 1. Introduction

Unmanned aerial vehicles (UAVs) provide new alternatives to traditional satellite-based remote sensing for obtaining high-resolution images in real time for precision agriculture and environmental applications [1,2]. When compared with other remote sensing platforms, UAVs have the advantage of being more flexible, lower cost, more independent of climatic variables [1], and they can provide higher-resolution information [3]. Therefore, these platforms offer appropriate resolution for vegetation observation which was not possible with traditional platforms. Different types of sensors, such as RGB digital cameras [4], thermal cameras [5], multispectral and hyperspectral cameras [6], and other sensors [7], allow for the extraction of agriculturally-useful information [8]. UAVs have been used to predict several crop characteristics, such as water status variability, crop region and tree crown mapping, vegetation index calculation, and species phenotyping, among others [5,9,10,11]. Estimating crop yields is one of the main challenges UAV-based vegetation analysis face today. With this aim the successful tomato detection using UAV images carried out by [12] seems promising. Additionally, the use of thermal information obtained from these devices in agricultural applications has been proposed, mostly focused on crop phenotyping under stress conditions [13,14,15,16,17]. However, depending on the thermal sensor used, thermal calibration is a crucial problem to be solved as uncooled sensors’ temperature measurements are constantly changing [18]. Research on the correct use of thermal cameras in agricultural applications is becoming more frequent, primarily for the development of studies showing the possibilities of using this equipment in crop monitoring. Rud et al. [19] stated that the use of images generated by thermal cameras is a very effective tool in assessing water availability in the cultivation of potatoes. Möller et al. [20] stated that the use of thermal cameras together with the use of digital cameras provide very good accuracy in determining physiological data for vineyards. DeJonge et al. [21] in studies with maize plants, affirmed that the monitoring and quantification of water stress through evaluating the canopy temperature by using thermometry can be useful in the detection of plant stress.

The use of thermal information is a remote sensing technique that is being developed to interpret the state of crops, the detection of pests and diseases related to the moisture content, and the determination of the energy balance and, therefore, the water needs, through the use of data obtained from thermal and multispectral cameras. Energy balance methods generally demand surface reflectance data detected remotely in the visible and near infrared regions of the spectrum to determine the thermal and infrared band [22,23,24]. Some of these models are described below:

1 The Surface Energy Balance Index (SEBI), developed by [25], is based on the idea of the Crop Water Stress Index (CWSI) and an essential aspect is the variation of the surface temperature with respect to the air temperature. It is a pioneering and widely-used model.
2 The two-source model (TSM), described in [26], is widely used, emphasizing its use in the case of the vineyard.
3 Clumped (three-source model: transpiration of the cover, evaporation from the soil of the row, evaporation from the ground between rows), generated from the works of [27], has been used in vineyards with good results, although it the accuracy of some parameters need to be improved (characterization of the roof architecture or parameterization of soil moisture) [28].
4 Surface Energy Balance Algorithm for Land (SEBAL), one of the most used models, developed by [23], calculates evapotranspiration as a residue of the energy balance of the surface. Within the most used models, SEBAL is designed to calculate the energy balance components, both locally and regionally, with minimum soil data [29,30].
5 The Simplified Surface Energy Index (S-SEBI) is a method based on a simplification of SEBI [25]. It is based on the contrast between a maximum and minimum surface reflectance temperature (albedo) for dry and wet conditions. Thus, it divides the available energy into sensible and latent heat flows. If the maximum and minimum surface temperatures are clearly available in the image, it does not require additional meteorological data, which becomes an advantage.
6 The Surface Energy Balance System (SEBS) is a SEBI modification to estimate the energy balance on the surface [31]. SEBS estimates the sensible and latent heat fluxes from satellite data and commonly-available meteorological data (air temperature and wind speed).
7 The Mapping Evapotranspiration at High Resolution and with Internalized Calibration (METRIC) is a widely-used model and proposes the modification of some parameters of the SEBAL model [22,32]. It is calibrated internally with the inclusion in the images of two reference surfaces (dry or wet pixels and hot or cold pixels) that permits fixing the boundary conditions in the energy balance and simplifying the need for atmospheric corrections.
8 The Surface Energy Balance to Measure Evapotranspiration (MEBES) is a development of SEBAL performed by [33] for application in a wide area of Spain. MEBES is a version developed for applications in regions where the availability of meteorological data is limited (incomplete data). MEBES was also validated with a lysimetric measurement at the local level. In addition, local actual evapotranspiration values (ETa) were compared using the Penman-Montieth method.
9 Remote Sensing of Evapotranspiration (ReSET) is a SEB model, proposed by [34] on the same principles as METRIC and SEBAL, but with some improvements, such as being able to integrate data from different meteorological stations.

These models each have their advantages and disadvantages [30], but they allow knowing ETa with different irrigation and cultivation management strategies [35], being able to approximate many parameters that determine the criteria for irrigation scheduling [36]. With the use of these methodologies, and based on thermometry parameters, the water status of the crop and the stress can be obtained. Many works are available for different herbaceous or woody species with different spatial scales but, in general, they are focused on the study of wide territories as support systems to the management of water resources [29,33,37,38].

All these models are based on the fact that the temperature of the canopy is an indicator of the water status of the plant, which is linked in turn to the stomatal conductance. Various water stress indices of crops have been developed based on the temperature of the canopy. The crop water stress index (CWSI) was developed by [39] and is increasingly being used to decide on irrigation management [40]. Other indicators are being implemented and applied to try to improve irrigation management.

To develop a functional methodology that utilizes images acquired with thermal cameras on UAVs, it is imperative that these sensors provide quantitative temperature information and that this temperature is measured with high precision, which demands a proper radiometric calibration. Another important problem related to the use of thermal images is the mosaicking in the photogrammetric process due to the low contrast of this type of image. This fact causes failures of the algorithms used for the automatic detection and pairing for the relative orientation of the images.

Thermal cameras are used in a wide range of different applications and, compared with conventional sensors, they do not depend on any external energy source [41]. These devices can be classified according to the type of image detector that they have, being classified as cooled or uncooled. Cooled thermal infrared cameras have the largest use in remote sensing because this type of camera is very sensitive and accurate [42], but the use of cooled sensors has some problematic because they are large, expensive, and consume a large amount of energy. Due to this fact, cooled thermal cameras are not usually mounted on small UAVs [42,43]. In contrast, the use of uncooled cameras coupled to UAVs is viable because they are lighter [5]. One disadvantage of the use of uncooled sensors is that these microbolometers are not as sensitive and accurate as in cooled systems and the majority of them are not calibrated, being only able to measure relative temperatures of a scene (image). For most remote sensing applications, accurate surface temperatures are required, which demands a calibrated thermal camera from the spectral and geometric point of view [5,43].

In uncooled thermal cameras, which mount thermo-electric cooler (TEC)-less microbolometers, the microbolometer is not stabilized to a constant temperature. This fact makes the sensor temperature fluctuate along with the temperature of the camera, which should be taken into account in any camera calibration model used [44]. Additionally, there are other causes that demand a calibration of TEC-less infrared sensors [44]:

1 Non-uniformity correction, which refers to the different operating points of the individual pixels of a microbolometer. A smoothing process is typically carried out in the current uncooled thermal cameras which attempts to equalize their performance.
2 Defective pixel correction, which refers to pixels that either do not work or whose parameters vary greatly from the mean. This is a characteristic of the sensor, which should be specified by the manufacturer. The correction of these pixels is based on their location and their interpolation based on the data obtained from neighbouring pixels. The main objective of this correction is to have a high-quality visual image rather than a high-quality radiometric value.
3 Shutter correction, which refers to the correction required due to the radiance of the camera interior that also varies with sensor temperature. Current uncooled thermal cameras perform an automatic shutter correction based on the time or change in sensor temperature.
4 Radiometric calibration, which refers to establishing the relationship between the response of the sensor and the temperature of the object. It is possible to approximate the sensor output signal with a Planck curve.
5 Temperature dependence correction, which refers to the effect of the sensor temperature on the response of the sensor. A linear correction that considers the signal from the object and the signal from the camera (dependent on camera temperature) is typically used to perform this type of correction.

In current uncooled thermal cameras, the first three sources of inaccuracies are corrected by the firmware included in the system. The fourth correction in devices is corrected by the digital acquisition system, if included in the sensor. However, the fifth correction is not usually performed, which leads to errors in the temperature measurements that are not acceptable for many applications, such as some agricultural or environmental applications. We found that the digital response of the camera is affected by the camera temperature in a non-linear manner. Thus, we will compare linear and non-linear classical models (polynomial models) to calibrate the thermal camera. Also, we will implement Artificial Neural Networks models to calibrate the camera because of their appropriate performance for solving highly non-linear problems [45].

Other sources of inaccuracies in thermal measurements using TEC-less cameras are [46]: size-of-source effect, distance effect, and environmental effects, among others, which are also present in the cooled cameras.

The procedure of generating the mosaic of orthoimages solves the general method of photogrammetry from the original images, obtaining the effect of obtaining an orthogonal perspective and covering the entire study area, which extends the field of view of the cameras [47] without introducing undesirable lens deformation [48]. Different types of photogrammetry software, such as PhotoScan^®^ (Agisoft, St. Petersburg, Russia), Pix4D^®^ (Pix4D, Lausanne, Switzerland), Apero-MicMac [49], VisualSfM [50], and Bundler [51], are used to obtain geomatic products from UAVs, such as georeferenced orthoimages and digital elevation models (DEM). These software packages require adequate image quality to obtain accurate geomatic products with the photogrammetry process.

Some problems related to obtaining orthoimages from thermal imaging are as follows: (1) the spatial resolution of commercial thermal chambers is still low, with commercial product resolutions varying from 160 × 120 to 1280 × 1024 [41], (2) vanadium oxide microbolometers have a higher noise level compared to other sensors [52], (3) compared to optical images, thermal images have low resolution and weak local contrast [53], and (4) in the particular case of non-refrigerated cameras, the acquisition of a time series of thermal images may fluctuate due to changes in sensor temperature [54]. Another problem of the use of thermal images in the photogrammetric process is that the images present very low contrast due to the low variation of the temperature in the observed objects. The low contrast of the images makes it difficult for computer vision algorithms to detect key points automatically. This problem implies that there are not enough matching points and the relative orientation process, with or without autocalibration, is not able to orient the entire set of images so that the mosaic of images, if it is achieved, has outstanding imperfections. In addition, it can lead to imprecision in the geometric calibration process of the camera.

In order to minimize these problems, filters have been developed for the treatment of images. These filters, in a simplified way, are described as part of mathematical procedures that consist of isolating the components of interest, so as to reinforce or soften the spatial contrasts of the grey level that integrate an image. It is performed by transforming the original grey levels of each pixel in such a way that they increase the difference with their corresponding neighbours. The filters can be classified according to the effect they produce to the images, being able to be of low step, of high step, of median, or directional, among others [55]. The purpose of the application of these filters in the context of the problem under study is to facilitate the photogrammetric procedure, primarily in the finding of tie-points.

Studies related to the pre-processing of images through the use of filters relate their functionality and effects in the images. Kou et al. [56] developed a detail enhancement algorithm to produce a detailed image, whereby the fine details can be amplified by enlarging all the gradients in the source image, except those of the pixels at the edges. From fine detail enhancement algorithms, fine details can be improved while avoiding halo artefacts and gradient inversion artefacts around the edges. Guidi et al. [57] analysed the effects of optical preprocessing with polarizing filters and digital preprocessing with high dynamic range (HDR) images to improve the conduction of 3D automated modelling based on Structure from Motion (SfM) and image matching. However, these authors observed that the metallic object does not preserve the polarizations of light and, consequently, is not affected by such an improvement. HDR-based techniques have also been analysed, revealing a moderate improvement in the ceramic object tested, on the order of 5%, compared to the standard images, but definitely a better result in the metal object (+63%).

Within this context, the objective of this work is to develop a procedure and an algorithm, based on machine learning, for radiometric calibration of uncooled thermal cameras. In addition, thermal image filtering to improve the photogrammetry solution is evaluated.

## 2. Materials and Methods

To obtain accurate high-resolution thermal products usable in precision agriculture, an integrated methodology is proposed. This methodology focuses on the accuracy of the acquired data, through the implementation of a novel calibration process, together with the appropriate data treatment during the mosaicking process to avoid post-processing artefacts.

### 2.1. Utilized Equipment

**Figure 1:**
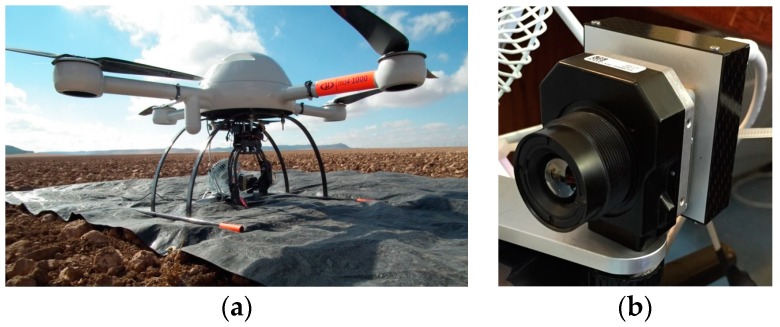
Unmanned aerial vehicle, Microdrone MD4-1000 (**a**) and uncooled thermal camera (**b**).

Different types of aircraft have different capabilities, with advantages or disadvantages depending on the application [1]. Compromises must be made between ease of flying, stability against wind, handling flight failures, distance covered, load capacity, and take-off/landing requirements. In this study, a Microdrone md4-1000 (Microdrones, Inc., Kreuztal, Germany) was utilized. It is a vertical take-off and landing (VTOL) quadcopter aircraft (Figure 1). The acronym VTOL denotes the capability of a flight vehicle to take off and land again in the vertical direction without the need of a runway. It employs four rotors or propellers on vertical shafts, mounted on one level of the bodywork. The UAV size is 1.030 m from motor to motor.

Regarding the flight performance, this UAV has a maximum rate of climb of 7.5 m∙s^–1^, a maximum horizontal speed of 12 m∙s^–1^, a maximum take-off weight (MTOW) of 6 kg, and a maximum load of 1.2 kg. The autonomy of the UAV reaches 40 min under optimal climatic conditions thanks to the 6S2P LiPo, 22.2 V, 13,000 mAh battery. With these characteristics, it can fly an area of approximately 80 ha per flight using a sensor that weights 0.3 kg.

The reference targets used to analyse the error of the geomatic products, especially designed for thermal applications as described below, were measured using Leica System 1200 GNSS receivers (Leica Geosystems AG, Wetzlar, Germany), which are dual-frequency systems that receive data from Global Positioning System (GPS) and Global Navigation Satellite System (GLONASS) constellation satellites and allow for measurement in a real-time kinematic (RTK) mode while static observations are recorded at the base receiver. During the subsequent post-processing step, the coordinates of the measured points were obtained in a global system with centimetre-level accuracy. This equipment updated its position with a frequency of 20 Hz (0.05 s) to minimize the possibility of recording false coordinates. The accuracy in RTK mode was 0.02 m in the *X* and *Y* axes, and 0.03 mm in the *Z* axis.

The thermal camera used to obtain the thermal images was the FLIR Tau2 (FLIR Systems, Inc., Wilsonville, OR, USA) (Figure 1), the main features of this camera are a focal of 9 mm (FOV 69° × 56°); an uncooled microbolometer of 640 (H) × 520 (V), a pixel size of 17 μm; a spectral band ranging 7.5–13.5 μm, and a weight of approximately 72 g without a lens.

### 2.2. Radiometric Calibration Data Acquisition

**Figure 2:**
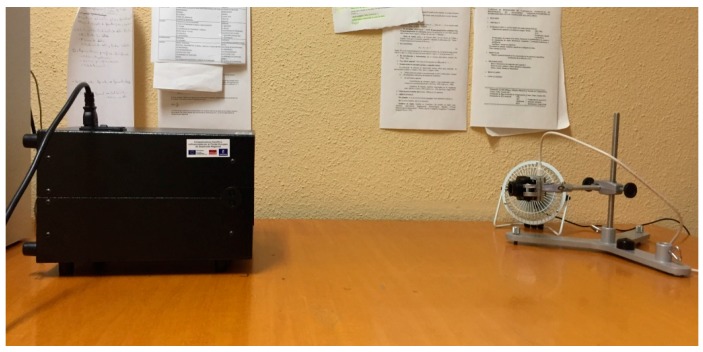
Disposal of the blackbody and the camera for calibration.

**Figure 3:**
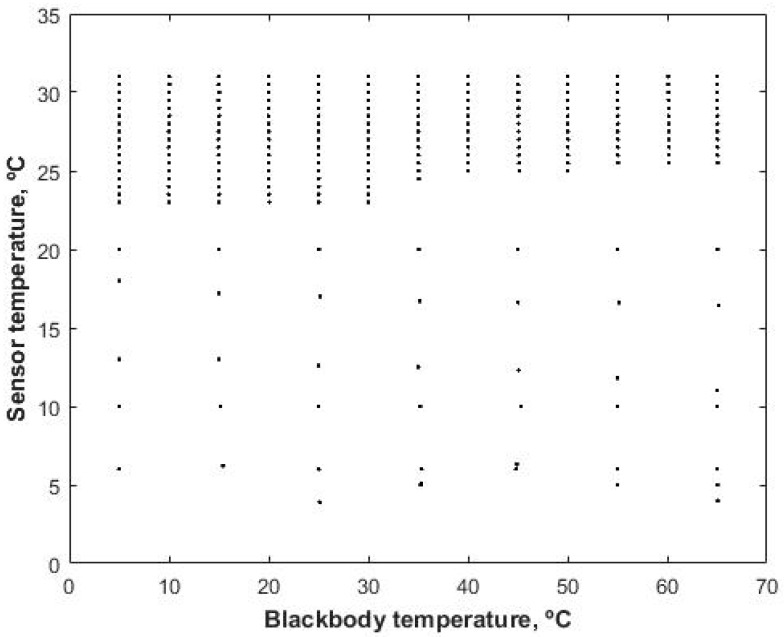
Range of thermal camera temperature and blackbody temperature analysed.

A radiometric calibration of the thermal cameras was conducted using a blackbody source Hyperion R Model 982 (Isothermal Technology Limited, Pine Grove, Southport, Merseyside, UK), with a large of 50 mm in diameter and 150 mm deep. The temperature range of the blackbody is from −10 to 80 °C. The thermal camera was installed in a fixed position against the black body at a distance of 0.5 m (Figure 2). The temperature of the sensor was measured and recorded for each image acquisition event. Blackbody temperatures used in the calibration process ranged from 5 to 65 °C in steps of 5 °C, which covers the range of temperatures found in agricultural applications. To obtain different values of the temperature of the camera, the experiment was carried out in a cooling room and in a regular room in which the temperature was modified to induce low and high temperatures with the cooling/heater system. Thus, a wide temperature range of the sensor is achieved to generalize the calibration model for any temperature condition. For each temperature of the blackbody images were obtained for sensor temperatures ranging between 5 and 31 °C (Figure 3).

A lower number of measurements were obtained for sensor temperatures lower than 20 °C, which corresponds with those obtained in the cooling room. Some problems related with water condensation in the black body and for reaching constant temperatures in the blackbody (it was set every 10 °C from 5 to 65 °C) made it a difficult task. With these data, it is ensured that a wide range of temperatures during the calibration process covers all the scenarios during agricultural applications. To ease the process of camera calibration a software called TermCal was developed by the authors in the MatLab^®^ (Mathworks, Natick, MA, USA) environment in which the user can select a representative area of the blackbody temperature captured by the thermal camera and determine the digital response of the image, assigning a value of sensor temperature and perform the camera calibration with the different models described in this paper.

### 2.3. Analyzed Algorithms for Radiometric Calibration

Three types of models were analysed in this paper to perform the camera calibration: (1) linear models (Equation (1)), (2) polynomial models (Equations (2)–(4)), which are the models that are traditionally applied for the correction for camera temperature variation [44], and (3) an artificial neural network [45], because of their adequate performance for solving highly non-linear regression problems. To determine the best model, 65% of the data were used for calibration and 35% for validation. The calibration and validation subsets were randomly selected ensuring that both data set cover the whole range of measurements:

TBB = p00 + p10 × DL + p01 × TC(1)

TBB = p00 + p10 × DL + p01 × TC + p20 × DL2 + p11 × DL × TC + p02 × TC2(2)

TBB = p00 + p10 × DL + p01 × TC + p20 × DL2 + p11 × DL × TC + p02 × TC2 + p30 × DL3 + p21 × DL2 × TC + p12 × DL × TC2(3)

TBB = p00 + p10 × DL + p01 × TC + p20 × DL2 + p11 × DL × TC + p02 × TC2 + p30 × DL3 + p21 × DL2 × TC + p12 × DL × TC2 + p03 × TC3(4)

where TBB is the temperature of the black body; DL is the digital response of the camera; TC is the temperature of the camera; and pij are the regression coefficients.

Artificial neural networks (ANNs) are mathematical models that simulate the functioning of a biological neuron, and these networks have some advantages which make their use possible in different fields of study. The training algorithm type used in the neural network was back propagation. Considering a neuron *j* in layer *i*, the sum of the input variables and corresponding weights (*S_j_*) in the input vector may be described according to Equation (5):

(5) $$S_{j}=w_{0j}+\sum_{i=1}^{n}w_{i}{}_{j}\cdot x_{I}$$

where *S_j_* is sum of the input variables with their corresponding weights in the neuron *j* of layer *i*; *w_ij_* is the weight associated with each of the input neurons with respect to the hidden layer nodes; *w*_0*j*_ is the weight associated with the first input neuron with respect to the hidden layer nodes; *x_I_* is the input value stored in each neuron *i*; and *n* is the number of input variables.

Equation (6) describes the output variable of neuron *j*:

(6) $$y_{j}=f(S_{j})$$

where *y_j_* is the output of neuron *j*; the activation function.

The activation function, for a hyperbolic tangent function responds to Equation (7):

(7) $$y_{j}=\tanh(S_{j})$$

where *y_j_* is the output of neuron *j*; and tanh is the hyperbolic tangent.

### 2.4. Analysis of Residuals

In order to analyse the model adjustments, the following statistics were utilized: number of observations (*n*); representing the amount of data to be evaluated. In this paper *n* = 266 images; the average values of the digital levels and the sensor temperature, which is given in Equation (8); the coefficient of determination (*R*^2^, Equation (9)); the root mean square error (RMSE), given by Equation (10); the relative error (RE, Equation (11)), and the similarity index (SI, Equation (12)).

(8) $$\bar{x}=\frac{\sum_{i=1}^{n}X_{i}}{n}$$

where $\bar{x}$ is the average of all observed values, *X_i_*, and *n* is the number of observations:

(9) $$R^{2}={\left[\sum_{i=1}^{n}\frac{\left(O_{n}-MO\right)\left(S_{n}-MS\right)}{\sqrt{\sum_{i=1}^{n}{\left(O_{n}-MO\right)}^{2}\sum_{i=1}^{n}{\left(S_{n}-MS\right)}^{2}}}\right]}^{2}$$

where *R*^2^ is the coefficient of determination; *O_n_* are the observed values; *S_n_* simulated values; *MO* is the average value of the *n* observed values; *MS* is the average value of *n* simulated values; and *n* is the number of observations:

(10) $$RMSE=\sqrt{\frac{\sum_{i=1}^{n}(S_{n}-O_{n}{)}^{2}}{n}}$$

where *RMSE* is the root mean square error (°C); *n* is the number of observations; *S_n_* are the simulated values; and *O_n_* are the observed values:

(11) $$RE=\left(\frac{RMSE}{MO}\right)\cdot 100$$

where *RE* is the relative error (%); *RMSE* is the root mean square error; and *MO* is the average value of the *n* observed values:

(12) $$SI=1-\left(\frac{\sum_{i=1}^{n}(S_{n}-O_{n}{)}^{2}}{\sum_{i=1}^{n}((S_{n}-MO)+(O_{n}-MO){)}^{2}}\right)$$

where *SI* is the similarity index; *n* is the number of observations; *S_n_* are the simulated values; *O_n_* are the observed values; and *MO* is the average value of the *n* observed values.

In addition to these statistics, error analysis will be performed by: (1) analysis of linear regression between observed and simulated values; (2) adjustment of the residuals to the normal distribution; (3) homoscedasticity; and (4) Cook’s distance for outlier detection.

### 2.5. Photogrammetry Process and Image Filtering

The procedure to acquire georeferenced geomatic products includes: (1) flight planning, which considers photogrammetric data that are provided by flight-planning software; (2) locating ground control points (GCPs) along the observed area; (3) measuring GCPs with a GNSS-RTK; (4) executing flights that follow the uploaded flight plan; (5) visually selecting the best set of images for post-processing by removing any blurred images; (6) entering the images and coordinates of the targets into photogrammetry software, which will self-calibrate the camera with the bundle-adjustment method; and (7) obtaining a georeferenced orthoimage, dense point cloud, and digital terrain model (DTM) [8,58].

**Figure 4:**
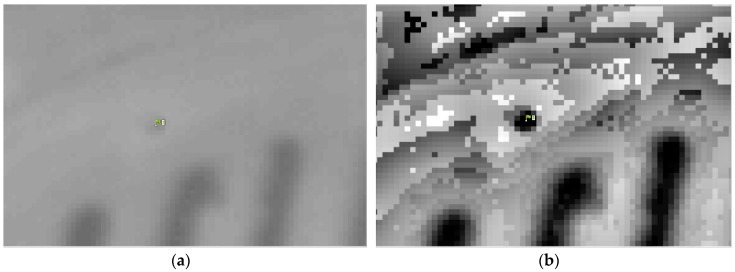
Example of photointerpretation in non-filtered images (**a**) and filtered images (**b**).

As stated in the introduction section, the photogrammetry process using thermal images is a challenging task because of the lack of contrast in the images and the difficulty of locating the GCPs in the images. To solve the low contrast in the image we propose to apply the Wallis filter [59] on thermal imaging, which has been successfully applied in other cases with visible images when the scene presents low contrast [60,61,62]. This filter applies a contrast enhancement to each zone of the image by adjusting the brightness values in specific areas of the image to make the measurement and the standard deviation match the user default values. This improvement achieves a good local contrast throughout the image (Figure 4a,b), which allows for better detection of key points and corresponding matching, as well as allowing the operator to improve the photointerpretation of the GCPs.

From the set of thermal images resulting from the planning flight, three sets of images are created: (1) the set of original thermal images, (2) the set of filtered thermal images after applying the Wallis filter to the set of thermal images, and (3) the set of radiometrically-calibrated thermal images after applying the thermal radiometric calibration to the original set of original thermal images. Photogrammetric processing was performed using Agisoft PhotoScan^®^ (Agisoft, St. Petersburg, Russia) software using the parameters described in Table 1.

For the processes of alignment of the images, determination of the dense cloud of points, and creation of the mesh, we used the set of thermal images treated with the Wallis filter. To texture and generate the final orthoimages, these images were replaced by the set of radiometrically-calibrated images. To apply the Wallis filter to the images, the GRAPHOS program was used [61]. In the process of self-calibration of the thermal camera with Agisoft PhotoScan^®^ software the following parameters were determined:

f: which is the focal length measured in pixels.
cx and cy: which are the coordinates of the main point.
b1, b2: which are the biased transformation coefficients.
k1, k2, k3, k4: which are the radial distortion coefficients.
p1, p2: which are the tangential distortion coefficients.

In addition to the results of the self-calibration procedure, errors were calculated in the GCPs.

The total error in *X*, *Y*, and *Z* was calculated from Equation (13):

(13) $$\mathrm{Total}\;\mathrm{error}\;=\;\sqrt{\left(\overset{n}{\underset{i=1}{\sum }}\frac{\left[\left(X_{i,\;est}-X_{i,\;in}\right)²+\left(Y_{i,est}-Y_{i,in}\right)²+\left(Z_{i,est}-Z_{i,\;in}\right)²\right]}{n}\right)}$$

where *X_i_* is the estimated value for the *X* coordinate for the position of the camera *i*; *X_i,in_* is the input value for the *X* coordinate for the position of the camera *i*; *Y_i,est_* is the estimated value for the *Y* coordinate for the position of the camera *i*; *Y_i,in_* is the value input for the *Y* coordinate for the position of the camera *i*; *Z_i,est_* is the estimated value for the *Z* coordinate for the position of the camera *i*; and *Z_i,in_* is the input value for the *Z* coordinate for the position of the camera *i*.

The photogrammetric processing was performed for three different situations: (1) use of the images as obtained from the camera with the factory settings, without filtering and without radiometric calibration; (2) use of filtered images without radiometric calibration; and (3) use of radiometrically-filtered and corrected images. The analysis of these cases allowed illustrating the improvements provided by the proposed methodology, by comparing the results in the photogrammetric processing of the images filtered and corrected radiometrically with those obtained with the same software when using the set of original thermal images (unfiltered and without radiometric calibration). In addition, the difference in temperature measurement was analysed by the use of radiometrically-corrected and uncorrected images.

The number of tie points obtained with the filtered and unfiltered images and the number of stereoscopic pairs generated were evaluated, which has a significant influence on the accuracy of the aerotriangulation with autocalibration and, consequently, on the quality of the obtained geomatic products.

### 2.6. Application to a Case Study

**Figure 5:**
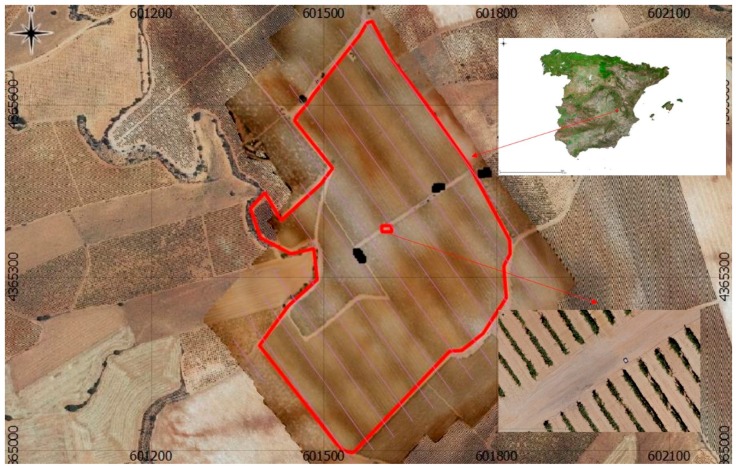
Location of the case study, and detail of the orthoimage obtained with the UAV. Black squares are ground measurements of validation points (PNOA, 2015).

The proposed methodology was implemented in a vineyard located in Iniesta (Cuenca, Spain) irrigated with water of the hydrogeological unit (H.U.) 08.29. (Figure 5). This unit is located in the southeast of Spain, on the eastern side of the La Mancha plains, with a total area of 8500 km^2^ and with relatively uniform agronomic features. This area is classified as semi-arid according to the aridity index (AI) described by [63] (AI = 0.36). Therefore, proper water management for irrigation is essential for rural development. Air temperature and other meteorological variables were recorded from an agro-meteorological station located 7 km from the plot. For the 2015 season, the precipitation was 268.4 mm year^−1^ and the annual reference evapotranspiration (ETo) was 1321.4 mm year^−1^.

The total area of the plot is 17.8 ha. The varieties cultivated in this plot were Sauvignon Blanc, Garnacha Tintorera, and Syrah. Plots were irrigated using drip irrigation systems, applying an average of 800 m^3^ ha^−1^, which means a deficit irrigation due to water scarcity and water restriction in the area. The irrigation system is divided into 12 sectors. The cultivation techniques applied were considered typical practices for vineyards.

Black squares are the locations of the ground measurements, which were performed over rain-fed neighbouring vineyards (eastern squares), irrigated vineyards that were irrigated the night before the flight (cantered squares), and irrigated vineyards irrigated seven days before the flight. In addition, soil measurements were obtained in the three placements.

### 2.7. Flight Planning and UAV Data Acquisition

The objective of flight planning was to generate a navigation file that guides the UAV to automatically capture images, with proper overlapping and sidelapping, according to the requirements of the final product (mainly GSD) and the requirements of the photogrammetry workflow [58]. In this case of study, the GSD employed was 0.20 m.

Overlapping was established in 60% and sidelapping in 40%. To ensure these values, flight planning should consider the errors of GPS, camera angles, etc. Microdrone photogrammetric flight planning software (MFLIP) was developed by the authors in collaboration with the company ICOM 3D (Asturias, Spain) for UAV flight planning, introducing all of the photogrammetry parameters required for each flight. The main result of the flight planning is an ASCII file in which each line includes an order for the UAV. This file is copied into the microSD card on the UAV. In addition, it generates a database with the theoretical footprints of the images, overlapping, among other data, in SHP files that can be opened with QGIS or a similar program. This information is useful in the photogrammetry workflow.

**Figure 6:**
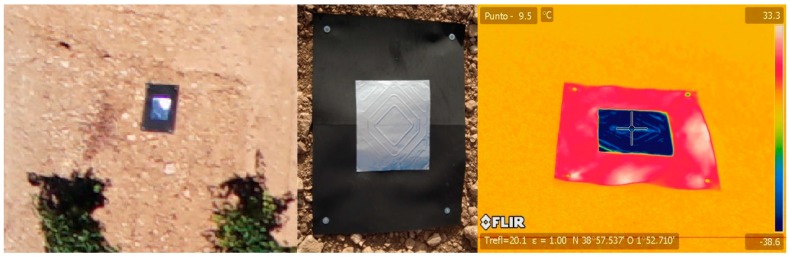
Type of GCPs used in thermal images.

With the dual objective of georeferencing the generated geomatic products and geometrically calibrating the camera lens, a total of eight GCPs were located and measured by GNSS-RTK. In order to allow the measurement of the GCPs in the thermal images, an evaluation was made of combinations of materials suitable for making the thermal targets, with EVA rubber material of dimensions of 0.60 × 0.80 m being chosen for their manufacture, with a polished aluminum plate of 0.34 × 0.29 m (Figure 6) in the centre. The polished aluminum sheet allows reflecting of the temperature of the sky, which is obviously very low compared to the temperature of the ground. Thus, it was easy to detect the targets in the thermal images by means of photointerpretation.

### 2.8. Ground Temperature Acquisition for Validation

In order to validate the proposed methodology, different temperature measurements were taken in different vines and soils just after the flight. To determine water stress in the plants, thermal measurements were obtained in different sectors. As the irrigation interval was one week, measurements were taken in vines irrigated from one week before the flight (five measurements in sector 3) to the night before (five measurements in sector 9). Additionally, neighboring rain-fed vines (five measurements) and soil (five measurements) were obtained. The measurements were made in the field with a FLIR B660 refrigerated thermal camera (FLIR Systems, Inc., Wilsonville, OR, USA). The emissivity was set to 1 to be able to compare both measurements. A Leica Zeno GNSS-RTK handheld system (Leica Geosystems AG) was used to capture the coordinates of each of the selected temperature sample points immediately after the flight, as well as the plants and the soil, to locate the sampled points in the generated orthoimagery. These temperature values were compared with the temperature values obtained from the orthoimages generated from the photogrammetric procedure using the radiometrically-corrected thermal images.

## 3. Results

### 3.1. Error Analysis of the Uncooled Thermal Camera

We obtained a series of data from 266 images of this camera that were analysed as they were captured with the configurations of the manufacturer. These same images were used in the comparison of the adjustments of the lineal and polynomial models and the ANN. Table 2 shows the statistics that determine the measuring accuracy of the camera. It can be observed that the measurement accuracy with the default parameters of the camera is very low, with an RMSE of 3.55 °C and an average RE of 8.47%. When using artificial neural networks, it can be observed that the RMSE decreased up to 1.37 °C and a RE of 8%.

**Figure 7:**
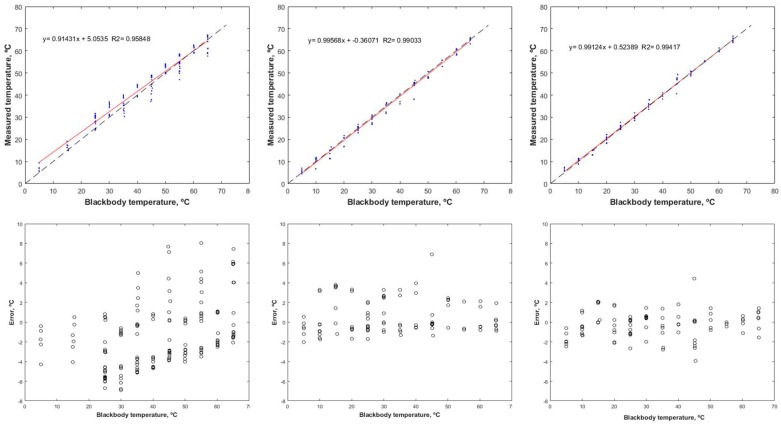

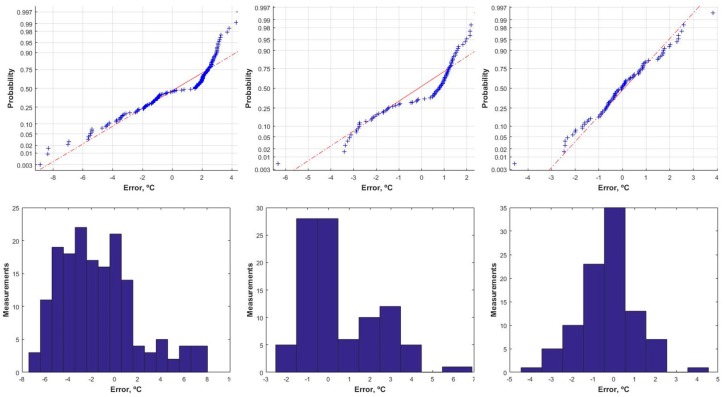
Error analysis for the FLIR Tau2 9 mm camera with the original configuration (column 1), the polynomial 2 model (column 2), and the artificial neural network model (column 3).

It can be observed that the RMSE decreases drastically from the manufacturer configuration to the polynomial and even the linear model (from 3.55 °C to 1.49 °C). According to the manufacturer, this camera should have an expected final precision of ±2 °C in absolute temperature measurements. There is an increase of the RMSE for polynomial models 3 and 4, which could be due to overfitting of the model. However, these differences are very slight. Thus, in this case, the best classical model is the polynomial 2 with a RMSE of 1.49 °C. However, when applying the ANN model, a RMSE of 1.37 °C can be reached. The slight difference does not justify the use of complex models based on machine learning over traditional polynomial models. However, a deep analysis of the model’s residuals should be performed to select the best model. Figure 7 shows the error analysis for the manufacturer configuration, polynomial 2 and ANN, respectively. It can be seen that the measurement errors are very high and are not randomly distributed, being non-homoscedastic. These errors call into question the usefulness of the camera in its original configuration for agronomic applications and demonstrate the need for an adequate calibration process to increase the accuracy of the measurement.

In agronomic applications, using satellite-derived data and non-local meteorological data, it was observed that a 2 °C error in soil temperature (Ts) corresponds to an error of 0.23 in the latent heat flux (LE), a 1 °C error in canopy temperature (Tc) corresponds to an error of 0.10 °C in LE for satellite data, an error of 1 °C in Ts corresponds to an error of 0.11 in LE, and an error of 0.5 °C in Tc corresponds to an error of 0.06 °C in LE for non-local meteorological data [64]. The RMSE obtained after calibration (1.4 °C) could be acceptable for agronomic applications, while using the manufacturer configuration (3.55 °C) could lead to gross errors in the energy balance model. Therefore, it is essential to perform an adequate calibration of uncooled thermal cameras.

Additionally, to implement the energy balance model from thermal images obtained with UAVs, it is recommended to perform a vicarious calibration with ground temperature measurements. Even if the camera is powered on before the flight for around 30 min and stabilized with climatic conditions, high variation of the sensor temperature could appear during the flight. Thus, the vicarious calibration could become a difficult task, requiring different points for ground measurement along the plot, increasing the cost of this activity. Here, we propose a calibration considering the sensor temperature and to perform a vicarious calibration in accessible, easy to measure points.

### 3.2. Results of Wallis Filter Application

The flight characteristics obtained from the photogrammetric process in Agisoft PhotoScan^®^ for each of the comparisons made are described in Table 3.

The number of images is the total number of images loaded in the project; the flight height is the average height above the terrain level calculated in the photogrammetric procedure in Agisoft PhotoScan^®^; ground resolution is the averaged field resolution on all aligned images; the coverage area is the size of the study area; the number of oriented images refers to the images for which the photogrammetric orientation has been corrected; projections is the total number of projections of valid tie-points; and re-projection error is the quadratic mean of the average of re-projection errors on all tie-points in all images. The re-projection error is the distance in the position in the image, in unit pixels, between the position detected by computational vision and the result of applying the collinearity equation from the position in the model system (terrain for relative or absolute orientation).

The results presented in Table 3 show that when applying the Wallis filter in the images the number of tie-points increases by 89% for the filtered images compared with unfiltered images. It is also observed that the number of projections increases when the filter is applied, with an increase of 63%. Table 3 also shows that the re-projection error decreases after filtering the images. These results demonstrate that the application of the Wallis filter to the images allows for an improvement in the processing of the images by the greater number of image tie-points recognized in the photogrammetric software used.

**Figure 8:**
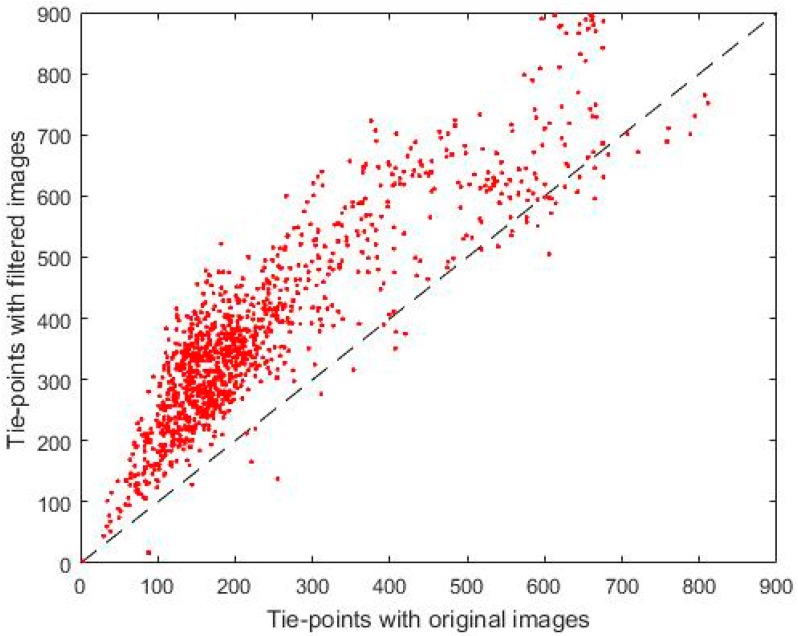
Comparison of the tie-points of the filtered images and the original images.

The results obtained from the detection of points of interest (key points) and their correspondences (points of connection or matching) on the images with the Wallis filter have greatly improved the results. Figure 8 shows a comparison between the number of tie-points in the alignment process using the original images (*X*-axis), and the number of coincident points for the filtered images (*Y*-axis).

**Figure 9:**
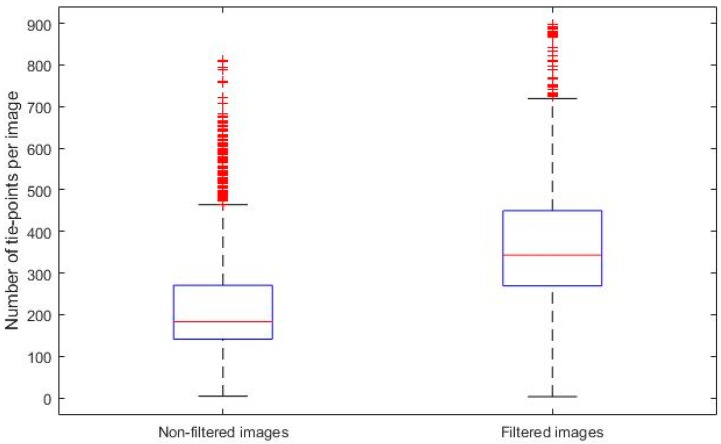
Box and whisker diagram of the number of link points of unfiltered and filtered.

To evaluate the statistical significance of the difference, a box and whisker diagram was obtained (Figure 9), which resulted in a significant difference.

**Figure 10:**
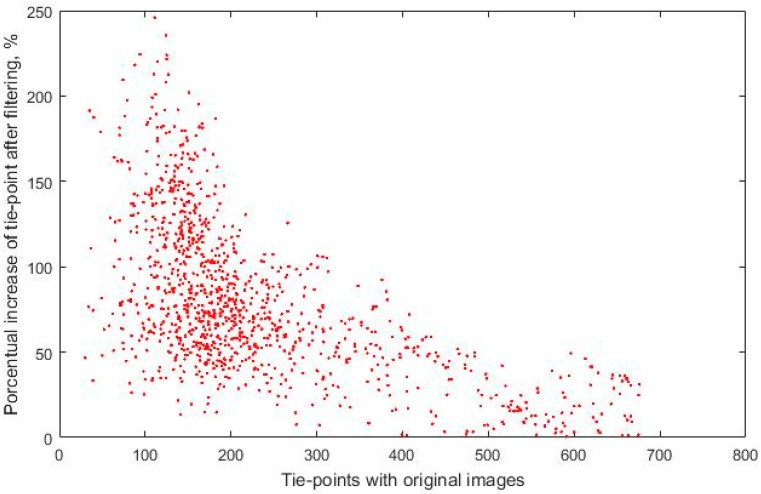
Increase in coincident points after Wallis filter application.

In addition, Figure 10 shows how the percentage of the increase of the tie-points resulting from the filtering process is greater for those images with a low number of points, a key question that allows the orientation of these images and explains that they may not be oriented if this technique is not applied. In this way, the effectiveness of the Wallis filter is confirmed in the automatic detection of tie-points for thermal imaging, which results in greater precision in the geometric calibration of the camera and in the relative and absolute aerotriangulation, which translates, finally, in high-quality geomatic products.

The calculated errors in *X*, *Y*, and *Z* are shown in Table 4 and Table 5, showing that the accuracy obtained in the GCPs after the absolute orientation is better for the filtered and geometrically-calibrated images, with reductions of 2.66 to 0.60 m in *X*, 2.45 to 0.43 m in *Y*, and from 6.19 to 0.98 m in *Z*. The 3D error reaches 7.2 m if the filtering process is not implemented and is reduced to 1.2 when filtering. The main error is obtained in *Z*, which means that the geomatic product obtained is accurate in planimmetry, but innacurate in altimetry. Thus, only the thermal orthoimage is a usable product while the dense cloud cannot be used to obtain the canopy volume. Thus, an interesting solution would be installing together multispectral, RGB, and thermal cameras to obtained different products on the same flights.

**Figure 11:**
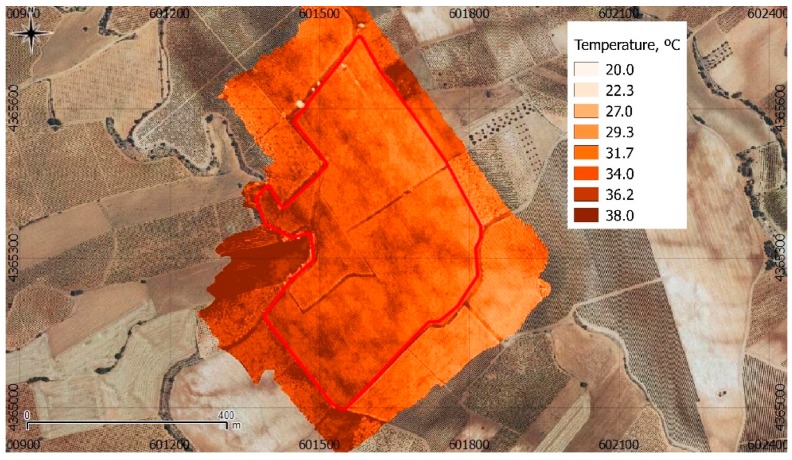
Ortoimage generated using calibrated and filtered images.

The geomatic product obtained presents a better quality besides being well georeferenced, as can be observed in Figure 11.

In some areas, the temperature difference between the corrected and uncorrected orthoimages reaches −5.06 °C and up to 1.93 °C.

### 3.3. Results of Temperature Measurements in the Case Study

Table 6 shows the comparison of three sources of information for temperatures in the validation surfaces: the measurements with the FLIR B660 hand-held thermal camera performed after the thermal flight as ground truth, those obtained by measurement in the uncalibrated images, and those obtained in the images resulting from the thermal calibration, both obtained from the generated orthoimages. The measurements, as described in the methodology (Figure 5), were obtained in neighbouring rain-fed vineyards (RV), vineyards irrigated the day before the flight (IV), vineyards irrigated seven days before the flight (7d-IV), and in the soil. Images were obtained for every condition.

**Figure 12:**
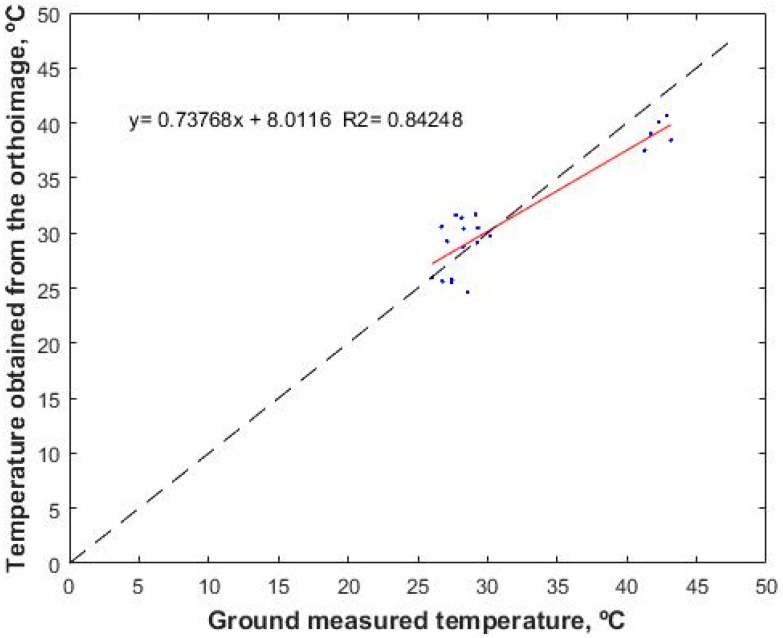
Comparison of the data obtained in the field with the data obtained from the generated geomatic product.

Figure 12 shows the adjustment of the data measured with the hand-held thermal camera and the calibrated camera using the procedure developed and the ortho-mosaic process described. It can be observed that the adjustment between the values is not adequate, with an RMSE of 2.6 °C, a maximum error of 4.7 °C and a SD of 2.7 °C. These errors can be due to: (1) the photogrammetric process of orthoimaging changes the temperature values in each pixel due to the need to apply an operation similar to the resampling method, which implies that the value assigned to each pixel of the final orthoimaging is the result of an average of close values of the different images involved, not allowing the software employed to request the use of the neighbour method closest to the optimal image, which would be ideal according to the principles of remote sensing; (2) the handheld camera is not the best method to perform a vicarious calibration, since its measurement accuracy, although markedly greater than the uncooled sensor on the UAV, is not less than 1 °C. Thus, in future studies we will use a spectroradiometer that measures in the thermal bands. Additionally, a method for selecting the more nadiral image of the set of images that measures the same point will be implemented. Thus, the temperature will be obtained from this image and not from the orthoimage.

## 4. Conclusions

To perform accurate measurements of temperature with uncooled thermal cameras it is necessary to perform and adequate camera calibration that considers the effect of the sensor temperature on the measurement. Additionally, a proper photogrammetry process should be implemented to generate high-quality mosaics from low-contrast thermal images. The proposed calibration procedure of uncooled thermal cameras, based on the use of artificial neural networks, which considers as input data the digital level of each pixel and the sensor temperature, noticeably improve accuracy. The temperature of the sensor has a great effect on the measurement accuracy of the thermal sensor. Without calibration errors, close to four degrees can be obtained while calibrating with the proposed methodology the measurement error can be reduced to approximately 1.5 °C.

The application of the Wallis filter to thermal imaging significantly improves the photogrammetric solution, providing a thermal high-quality thermal orthoimage. Similar problems have been found by other researchers that utilized similar cameras [65]. They found temperature differences between −5 °C to 20 °C depending of the time of the day. They performed vicarious calibration but they did not accounted for a laboratory camera calibration as the presented in this manuscript.

Applications based on temperature measurements with uncooled microbolometers should consider accurate camera calibration, proper systems for measuring microbolometer temperature, and a rigorous methodology to perform UAV flights that warranties the measurement precision. Also, images treatment in the photogrammetry workflow should carefully perform to avoid geometric inaccuracies. All these aspects, increase the cost for obtaining a reliable thermal product.

With this research, we detected the need to improve the quality of the vicarious calibration with accurate spectroradiometers and the need to obtain new methodologies to obtain temperature values from the best positioned image for each point. We will focus our future research in these two newly-detected lines of research.

## Figures and Tables

**Table 1:** Parameters used in Agisoft PhotoScan^®^.

**Alignment Parameters**	
Accuracy	High
Pair preselection	Generic
Key point limit	40.000
Tie point limit	4.000
Adaptive camera model fitting	yes
Optimized parameters	f, b1, b2, cx, cy, k1–k4, p1, p2
Dense point cloud	
Quality	Medium
Depth filtering	Mild
**Model**	
Surface type	Arbitrary
Source data	Dense cloud
Face count	High
Interpolation	Enabled
Orthomosaic	
Mapping mode	Orthophoto
Blending mode	Mosaic

**Table 2:** Main statistic indices of the manufacturer configuration, linear model, polynomial models (P1–P4), and the artificial neural network model.

	Manufacturer Configuration	Linear	P1	P2	P3	P4	ANN
Data	266	95	95	95	95	95	95
R^2^	0.96	0.99	0.99	0.99	0.99	0.99	0.99
RMSE, °C	3.55	1.81	1.81	1.49	1.51	1.51	1.37
Relative Error, %	8.47	5.59	5.57	4.59	4.66	4.66	4.22
Similarity Index	0.99	1.00	1.00	1.00	1.00	1.00	1.00

**Table 3:** Characteristics of each of the photogrammetric procedures performed for each of the described situations.

	Unfiltered Images	Filtered Images
Number of images	1154	1154
Flight height (m)	81.1	80.4
Ground resolution (cm pix^−1^)	13.8	13.5
Covered area (km^2^)	0.366	0.364
Number of images oriented	1.148	1.151
Tie-points	58,193	110,089
Projections	272,078	445,291
Re-projection error (pix)	0.504	0.442

**Table 4:** Control points of images not filtered and not geometrically calibrated.

GCP	Error X (mm)	Error Y (mm)	Error Z (mm)	Total (mm)	Image (pix)
1	1.00	−1.12	0.80	1.70	0.58 (34)
2	−3.81	−0.33	2.78	4.73	0.97 (49)
3	4.82	1.65	−10.87	12.01	0.74 (32)
4	2.69	1.41	5.83	6.57	0.65 (30)
5	0.53	3.56	2.36	4.30	0.36 (32)
7	−2.41	−5.36	−6.14	8.50	0.56 (26)
8	−1.60	0.68	4.89	5.19	0.86 (24)
9	−1.48	0.33	−8.92	9.05	0.53 (31)
Total	2.66	2.45	6.20	7.18	0.70

**Table 5:** Control points of the filtered and calibrated geometrically images.

GCP	Error X (mm)	Error Y (mm)	Error Z (mm)	Total (mm)	Image (pix)
1	−0.37	0.46	−0.98	1.14	0.57 (34)
2	−0.19	−0.24	−0.04	0.32	1.65 (49)
3	0.02	0.32	0.13	0.35	0.64 (31)
4	0.05	−0.64	1.33	1.48	0.87 (32)
5	1.32	0.61	1.00	1.77	0.38 (32)
7	−0.98	−0.32	−1.78	2.05	0.67 (27)
8	−0.20	0.28	0.90	0.97	0.78 (23)
9	0.08	−0.39	−0.17	0.43	0.51 (31)
Total	0.61	0.43	0.98	1.23	0.92

**Table 6:** Results obtained from the points sampled.

	Handheld Camera		Original Configuration		Corrected Data	
	Mean	SD	Mean	SD	Mean	SD
RV 1	30.2	0.3	32.0	0.1	29.7	0.1
RV 2	29.2	0.2	33.8	0.0	31.7	0.1
RV 3	27.7	0.3	33.8	0.2	31.6	0.2
RV 4	29.3	0.3	32.7	0.4	30.5	0.4
RV 5	29.2	0.3	31.4	0.1	29.1	0.2
IV 1	27.4	0.1	28.3	0.0	25.8	0.0
IV 2	26.8	0.3	28.2	0.3	25.6	0.3
IV 3	28.6	0.2	27.2	0.0	24.6	0.0
IV 4	27.4	0.2	28.1	0.5	25.5	0.5
IV 5	26.0	0.2	28.5	0.3	26.0	0.3
7d-IV 1	28.2	0.3	33.4	0.0	31.4	0.0
7d-IV 2	26.7	0.3	32.7	0.3	30.6	0.3
7d-IV 3	28.3	0.1	32.5	0.2	30.4	0.2
7d-IV 4	27.1	0.1	31.4	0.1	29.3	0.1
7d-IV 5	28.3	0.1	30.9	0.0	28.8	0.0
Soil 1	42.8	0.2	42.2	0.1	40.7	0.1
Soil 2	42.3	0.3	41.6	0.1	40.1	0.1
Soil 3	41.7	0.3	40.6	0.1	39.0	0.1
Soil 4	43.2	0.5	40.1	0.1	38.4	0.1
Soil 5	41.3	0.2	39.2	0.0	37.5	0.0

## References

[1] Zhang C., Kovacs J.M. (2012). The application of small unmanned aerial systems for precision agriculture: A review. Precis. Agric..

[2] Ballesteros R., Ortega J.F., Hernández D., Moreno M.A. (2014). Applications of georeferenced high-resolution images obtained with unmanned aerial vehicles. Part II: Application to maize and onion crops of a semi-arid region in Spain. Precis. Agric..

[3] Laliberte A.S., Rango A. (2011). Image Processing and Classification Procedures for Analysis of Sub-decimeter Imagery Acquired with an Unmanned Aircraft over Arid Rangelands. GISci. Remote Sens..

[4] Majidi B., Bab-Hadiashar A. Real time aerial natural image interpretation for autonomous ranger drone navigation. Proceedings of the Digital Imag Computing Techniques and Application (DICTA 2005).

[5] Berni J.A.J., Zarco-Tejada P.J., Suárez L., Fereres E. (2009). Thermal and narrowband multispectral remote sensing for vegetation monitoring from an unmanned aerial vehicle. IEEE Trans. Geosci. Remote Sens..

[6] Zarco-Tejada P.J., Berjón A., López-Lozano R., Miller J.R., Martín P., Cachorro V., González M.R., De Frutos A. (2005). Assessing vineyard condition with hyperspectral indices: Leaf and canopy reflectance simulation in a row-structured discontinuous canopy. Remote Sens. Environ..

[7] Kingston D.B., Beard A.W. Real-time Attitude and Position Estimation for Small UAVs using Low-cost Sensors. Proceedings of the AIAA 3rd Unmanned Unlimited Technical Conference on Workshop and Exhibit.

[8] Ribeiro-Gomes K., Hernandez-Lopez D., Ballesteros R., Moreno M.A. (2016). Approximate georeferencing and automatic blurred image detection to reduce the costs of UAV use in environmental and agricultural applications. Biosyst. Eng..

[9] Baluja J., Diago M.P., Balda P., Zorer R., Meggio F., Morales F., Tardaguila J. (2012). Assessment of vineyard water status variability by thermal and multispectral imagery using an unmanned aerial vehicle (UAV). Irrig. Sci..

[10] Zaman-Allah M., Vergara O., Araus J.L., Tarekegne A., Magorokosho C., Zarco-Tejada P.J., Hornero A., Albà A.H., Das B., Craufurd P. (2015). Unmanned aerial platform-based multi-spectral imaging for field phenotyping of maize. Plant Methods.

[11] Senthilnath J., Kandukuri M., Dokania A., Ramesh K.N. (2017). Application of UAV imaging platform for vegetation analysis based on spectral-spatial methods. Comput. Electron. Agric..

[12] Senthilnath J., Dokania A., Kandukuri M., Ramesh K.N., Anand G., Omkar S.N. (2016). Detection of tomatoes using spectral-spatial methods in remotely sensed RGB images captured by UAV. Biosyst. Eng..

[13] Bellvert J., Marsal J., Girona J., Zarco-Tejada P.J. (2014). Seasonal evolution of crop water stress index in grapevine varieties determined with high-resolution remote sensing thermal imagery. Irrig. Sci..

[14] Bellvert J., Zarco-Tejada P.J., Girona J., Fereres E. (2014). Mapping crop water stress index in a “Pinot-noir” vineyard: Comparing ground measurements with thermal remote sensing imagery from an unmanned aerial vehicle. Precis. Agric..

[15] Elvanidi A., Katsoulas N., Bartzanas T., Ferentinos K.P., Kittas C. (2017). Crop water status assessment in controlled environment using crop reflectance and temperature measurements. Precis. Agric..

[16] Ortega-Farías S., Ortega-Salazar S., Poblete T., Kilic A., Allen R., Poblete-Echeverría C., Ahumada-Orellana L., Zúñiga M., Sepúlveda D. (2016). Estimation of energy balance components over a drip-irrigated olive orchard using thermal and multispectral cameras placed on a helicopter-based unmanned aerial vehicle (UAV). Remote Sens..

[17] Santesteban L.G., Di Gennaro S.F., Herrero-Langreo A., Miranda C., Royo J.B., Matese A. (2017). High-resolution UAV-based thermal imaging to estimate the instantaneous and seasonal variability of plant water status within a vineyard. Agric. Water Manag..

[18] Gómez-Candón D., Virlet N., Labbé S., Jolivot A., Regnard J.L. (2016). Field phenotyping of water stress at tree scale by UAV-sensed imagery: New insights for thermal acquisition and calibration. Precis. Agric..

[19] Rud R., Cohen Y., Alchanatis V., Levi A., Brikman R., Shenderey C., Heuer B., Markovitch T., Dar Z., Rosen C. (2014). Crop water stress index derived from multi-year ground and aerial thermal images as an indicator of potato water status. Precis. Agric..

[20] Möller M., Alchanatis V., Cohen Y., Meron M., Tsipris J., Naor A., Ostrovsky V., Sprintsin M., Cohen S. (2007). Use of thermal and visible imagery for estimating crop water status of irrigated grapevine. J. Exp. Bot..

[21] DeJonge K.C., Taghvaeian S., Trout T.J., Comas L.H. (2015). Comparison of canopy temperature-based water stress indices for maize. Agric. Water Manag..

[22] Allen R.G., Tasumi M., Morse A., Trezza R., Wright J.L., Bastiaanssen W., Kramber W., Lorite I., Robinson C.W. (2007). Satellite-based energy balance for mapping evapotranspiration with internalized calibration (METRIC)—Applications. J. Irrig. Drain. Eng..

[23] Bastiaanssen W.G.M., Menenti M., Feddes R.A., Holtslag A.A.M. (1998). A remote sensing surface energy balance algorithm for land (SEBAL). 1. Formulation. J. Hydrol..

[24] Gowda P.H., Chavez J.L., Colaizzi P.D., Evett S.R., Howell T.A., Tolk J.A. (2008). ET mapping for agricultural water management: Present status and challenges. Irrig. Sci..

[25] Roerink G.J., Su Z., Menenti M. (2000). S-SEBI: A simple remote sensing algorithm to estimate the surface energy balance. Phys. Chem. Earth Part B Hydrol. Ocean. Atmos..

[26] Norman J.M., Kustas W.P., Humes K.S. (1995). Source approach for estimating soil and vegetation energy fluxes in observations of directional radiometric surface temperature. Agric. For. Meteorol..

[27] Brenner A.J., Incoll L.D. (1997). The effect of clumping and stomatal response on evaporation from sparsely vegetated shrublands. Agric. For. Meteorol..

[28] Poblete-Echeverría C., Ortega-Farias S. (2009). Estimation of actual evapotranspiration for a drip-irrigated merlot vineyard using a three-source model. Irrig. Sci..

[29] Allen R., Irmak A., Trezza R., Hendrickx J.M.H., Bastiaanssen W., Kjaersgaard J. (2011). Satellite-based ET estimation in agriculture using SEBAL and METRIC. Hydrol. Process..

[30] Liou Y.A., Kar S.K. (2014). Evapotranspiration estimation with remote sensing and various surface energy balance algorithms—A review. Energies.

[31] Su Z. (2002). The Surface Energy Balance System (SEBS) for estimation of turbulent heat fluxes. Hydrol. Earth Syst. Sci..

[32] Allen R.G., Tasumi M., Morse A. (2005). Satellite-based evapotranspiration by METRIC and Landsat for western states water management. US Bur. Reclam. Evapotranspiration Workshop.

[33] Ramos J.G., Cratchley C.R., Kay J.A., Casterad M.A., Martínez-Cob A., Domínguez R. (2009). Evaluation of satellite evapotranspiration estimates using ground-meteorological data available for the Flumen District into the Ebro Valley of N.E. Spain. Agric. Water Manag..

[34] Elhaddad A., Garcia L.A., Chavez J.L. (2011). Using a surface energy balance model to calculate spatially distributed actual evapotranspiration. J. Irrig. Drain. Eng..

[35] Poblete-Echeverría C., Ortega-Farias S. (2012). Calibration and validation of a remote sensing algorithm to estimate energy balance components and daily actual evapotranspiration over a drip-irrigated Merlot vineyard. Irrig. Sci..

[36] Allen R.G., Pereira L.S., Raes D., Smith M. (1998). Crop Evapotranspiration: Guidelines for Computing Crop Requirements FAO Irrigation and Drainage Paper No. 56. FAO Rome.

[37] Awan U.K., Anwar A., Ahmad W., Hafeez M. (2016). A methodology to estimate equity of canal water and groundwater use at different spatial and temporal scales: A geo-informatics approach. Environ. Earth Sci..

[38] Mahmoud S.H., Alazba A.A. (2016). A coupled remote sensing and the Surface Energy Balance based algorithms to estimate actual evapotranspiration over the western and southern regions of Saudi Arabia. J. Asian Earth Sci..

[39] Idso S.B., Jackson R.D., Pinter P.J., Reginato R.J., Hatfield J.L. (1981). Normalizing the stress-degree-day parameter for environmental variability. Agric. Meteorol..

[40] King B.A., Shellie K.C. (2016). Evaluation of neural network modeling to predict non-water-stressed leaf temperature in wine grape for calculation of crop water stress index. Agric. Water Manag..

[41] Gade R., Moeslund T.B. (2014). Thermal cameras and applications: A survey. Mach. Vis. Appl..

[42] Sheng H., Chao H., Coopmans C., Han J., McKee M., Chen Y. Low-cost UAV-based thermal infrared remote sensing: Platform, calibration and applications. Proceedings of the 2010 IEEE/ASME International Conference on Mechatronic Embedded Systems and Applications (MESA).

[43] Jensen A.M., McKee M., Chen Y. Procedures for processing thermal images using low-cost microbolometer cameras for small unmanned aerial systems. Proceedings of the 2014 IEEE International Geoscience and Remote Sensing Symposium.

[44] Budzier H., Gerlach G. (2015). Calibration of uncooled thermal infrared cameras. J. Sens. Sens. Syst..

[45] Bishop C.M. (1995). Neural networks for pattern recognition. J. Am. Stat. Assoc..

[46] Mcevoy H., Simpson R., Machin G. Quantitative InfraRed Thermography Review of current thermal imaging temperature calibration and evaluation facilities, practices and procedures, across EURAMET. Proceedings of the 11th International Conference on Quantitative InfraRed Thermography (QIRT 2012).

[47] Botterill T., Mills S., Green R. Real-time aerial image mosaicing. Proceedings of the International Conference Image and Vision Computing New Zealand.

[48] Ghosh D., Kaabouch N. (2016). A survey on image mosaicing techniques. J. Vis. Commun. Image Represent..

[49] Pierrot-Deseilligny M., Clery I. Apero, an Open Source Bundle Adjusment Software for Automatic Calibration and Orientation of Set of Images. Proceedings of the ISPRS Symposium, 3DARCH11.

[50] Wu C. VisualSFM: A Visual Structure from Motion System. http://ccwu.me/vsfm/doc.html.

[51] Snavely N., Seitz S.M., Szeliski R. (2006). Photo tourism: Exploring Photo Collections in 3D. ACM Trans. Graph..

[52] Kastek M., Dulski R., Trzaskawka P., Bieszczad G. (2010). Sniper detection using infrared camera: Technical possibilities and limitations. SPIE Defense, Security, and Sensing.

[53] Hartmann W., Tilch S., Eisenbeiss H., Schindler K. (2012). Determination of the Uav Position By Automatic Processing of Thermal Images. ISPRS-Int. Arch. Photogramm. Remote Sens. Spat. Inf. Sci..

[54] Smoorenburg M., Volze N., Tilch S., Hartmann W., Naef F., Kinzelbach W. Evaluation of Thermal Infrared Imagery Acquired with an Unmanned Aerial Vehicle for Studying Hydrological Processes. Proceedings of the EGU General Assembly Conference Abstracts.

[55] Pérez Álvarez J.A. (2001). Apuntes de Fotogrametría II. Cent. Univ. Mérida.

[56] Kou F., Chen W., Li Z., Wen C. (2015). Content adaptive image detail enhancement. IEEE Signal Process. Lett..

[57] Guidi G., Gonizzi S., Micoli L.L. (2014). Image pre-processing for optimizing automated photogrammetry performances. ISPRS Ann. Photogramm. Remote Sens. Spat. Inf. Sci..

[58] Ballesteros R., Ortega J.F., Hernández D., Moreno M.A. (2014). Applications of georeferenced high-resolution images obtained with unmanned aerial vehicles. Part I: Description of image acquisition and processing. Precis. Agric..

[59] Wallis K.F. (1974). Seasonal adjustment and relations between variables. J. Am. Stat. Assoc..

[60] Gaiani M., Remondino F., Apollonio F.I., Ballabeni A. (2016). An advanced pre-processing pipeline to improve automated photogrammetric reconstructions of architectural scenes. Remote Sens..

[61] González-Aguilera D., López-Fernández L., Rodriguez-Gonzalvez P., Guerrero D., Hernandez-Lopez D., Remondino F., Menna F., Nocerino E., Toschi I., Ballabeni A. (2016). Development of an all-purpose free photogrammetric tool. ISPRS Int. Arch. Photogramm. Remote Sens. Spat. Inf. Sci..

[62] Jazayeri I., Fraser C. (2008). Interest operators in close-range object reconstruction. Int. Arch. Photogramm. Remote Sens. Spat. Inf. Sci. Beijing.

[63] (1997). UNEP World Atlas of Desertification.

[64] Sánchez J.M., Kustas W.P., Caselles V., Anderson M.C. (2008). Modelling surface energy fluxes over maize using a two-source patch model and radiometric soil and canopy temperature observations. Remote Sens. Environ..

[65] Torres-Rua A. (2017). Vicarious calibration of sUAS microbolometer temperature imagery for estimation of radiometric land surface temperature. Sensors.

